# Comparison of Robotic and Open Lobectomy for Lung Cancer in Marginal Pulmonary Function Patients: A Single-Centre Retrospective Study

**DOI:** 10.3390/curroncol31010009

**Published:** 2023-12-24

**Authors:** Carmelina Cristina Zirafa, Beatrice Manfredini, Gaetano Romano, Elisa Sicolo, Andrea Castaldi, Elena Bagalà, Riccardo Morganti, Claudia Cariello, Federico Davini, Franca Melfi

**Affiliations:** 1 Minimally Invasive and Robotic Thoracic Surgery, Surgical, Medical, Molecular, and Critical Care Pathology Department, University Hospital of Pisa, 56124 Pisa, Italy; beatrice.manfredini91@gmail.com (B.M.); gaetano.romano@ao-pisa.toscana.it (G.R.); e.sicolo@studenti.unipi.it (E.S.); a.castaldi@studenti.unipi.it (A.C.); e.bagala@studenti.unipi.it (E.B.); f.davini@ao-pisa.toscana.it (F.D.); franca.melfi@unipi.it (F.M.); 2 Section of Statistics, University Hospital of Pisa, 56124 Pisa, Italy; r.morganti@ao-pisa.toscana.it; 3 Cardiothoracic and Vascular Anaesthesia and Intensive Care, Department of Anaesthesia and Critical Care Medicine, University Hospital of Pisa, 56124 Pisa, Italy; c.cariello@ao-pisa.toscana.it

**Keywords:** robotic surgery, minimally invasive surgery, thoracotomy, marginal respiratory function, non-small-cell lung cancer, FEV1, postoperative complications

## Abstract

Background: The treatment of non-small-cell lung cancer (NSCLC) patients with reduced respiratory function represents a challenge for thoracic surgeons. Minimally invasive surgery seems to be beneficial for these patients because it reduces tissue trauma and its impact on respiratory mechanics. Application of the robotic technique, the use of CO_2_ insufflation and longer surgical time are factors that could influence the outcomes of marginal pulmonary function patients. The objective of this study was to evaluate the impact of the robotic technique on the postoperative outcomes of patients with poor lung function. Methods: We retrospectively collected and analyzed data from consecutive marginal respiratory function patients who underwent robotic or open lobectomy for NSCLC. Data regarding clinical, operative and postoperative details were compared between the open and robotic approaches. Results: The outcomes of 100 patients with reduced respiratory function were evaluated, of whom 59 underwent open lobectomies and 41 underwent robotic lobectomies. Robotic lobectomy was characterized by a longer operative time, a reduced hospital stay and a lower incidence of postoperative complications (22% vs. 33.9%), when compared to the open approach. Conclusion: Robotic lobectomy is a safe and feasible procedure for patients with marginal pulmonary function.

## 1. Introduction

Surgery with radical intent is currently considered the most effective treatment for resectable non-small-cell lung cancer (NSCLC). In detail, in terms of oncological outcomes, lobectomy is the treatment of choice for lung cancer patients with an adequate respiratory reserve who are candidates for surgery [1,2,3]. However, among lung cancer patients, those with reduced respiratory function represent a challenge for thoracic surgeons. Different diagnostic examinations are available to assess the respiratory function of patients eligible for lung resection. However, there is a lack of widespread consensus on the most commonly used and appropriate test for predicting postoperative risk [4,5]. Previous studies have evaluated the role of these different examinations to stratify the operative risk, also taking into account the recommendations from international guidelines. These studies revealed that, in clinical practice, Forced Expiratory Volume in the first second (FEV1) is the most commonly employed test [6]. 

FEV1 is indeed recognized as a predictor of postoperative complications and, in the case of planned pulmonary lobectomy, patients are eligible for surgery with an acceptable operative risk when FEV1 is >1.5 liters (L) [7]. The effect of preoperative pulmonary function on short-term outcomes after lung resection is well understood; however, a reduction in preoperative FEV1 is not associated with a poorer long-term prognosis. Furthermore, the onset of complications after pulmonary resection is also considered a negative predictor of overall survival in lung cancer patients. 

Otherwise, less invasive treatments, such as sublobar resections or stereotaxic irradiation, are often considered for preserving pulmonary function, to the detriment of oncological radicality. 

Following the introduction of minimally invasive surgery (MIS), numerous studies have demonstrated the benefits of performing lobectomy using a minimally invasive approach when compared to thoracotomy, such as fewer complications, a shorter postoperative hospital stay and similar oncological results [8,9,10]. Ideally, patients with poor respiratory function are expected to benefit from MIS due to the reduction in tissue trauma and its subsequent effects on respiratory mechanics, which can lead to better outcomes. Robotic surgery is considered the latest innovation in minimally invasive approaches and, over the last two decades, its application has increased in the thoracic field and has expanded to encompass challenging cases and high-risk patients. Evaluating the features of the robotic technique, the use of carbon dioxide (CO_2_) insufflation during the procedure and the longer operative time, due to docking/undocking of the system, are factors that could affect the postoperative results of patients with marginal pulmonary function. 

Currently, studies on the application of robotic surgery in patients with marginal lung function are limited. The objective of this study was to evaluate the impact of robotic surgery on the postoperative outcomes of patients with poor lung function.

## 2. Materials and Methods

The study design, patient enrolment and data collection methods were reviewed and approved by the institutional review board (ID of Ethics Committee: 19211). Informed consent was obtained from all patients enrolled in this study. 

The present study was written according to the Strengthening the Reporting of Observational Studies in Epidemiology (STROBE) statement.

Data from marginal pulmonary function patients who underwent robotic or open lobectomy with hilar/mediastinal lymphadenectomy between January 2014 and December 2019 were retrospectively collected and analyzed. 

Patients with a diagnosis of non-small-cell lung cancer, with marginal pulmonary function defined as an FEV1 < 1.5 L, who underwent lobectomy with curative intent were selected for this case–control study. Patients with a single adrenal or brain metastasis, who had already received treated at the time of surgical lung resection, were included in this study.

Patients who underwent non-anatomical lung resection, bronchoplasty, sleeve resection, or bilobectomy; concomitant chest wall resection; induction therapy; had poor cardiac reserve (based on cardiac function evaluation); or had other concurrent malignant diseases were excluded from this study.

**Figure 1:**
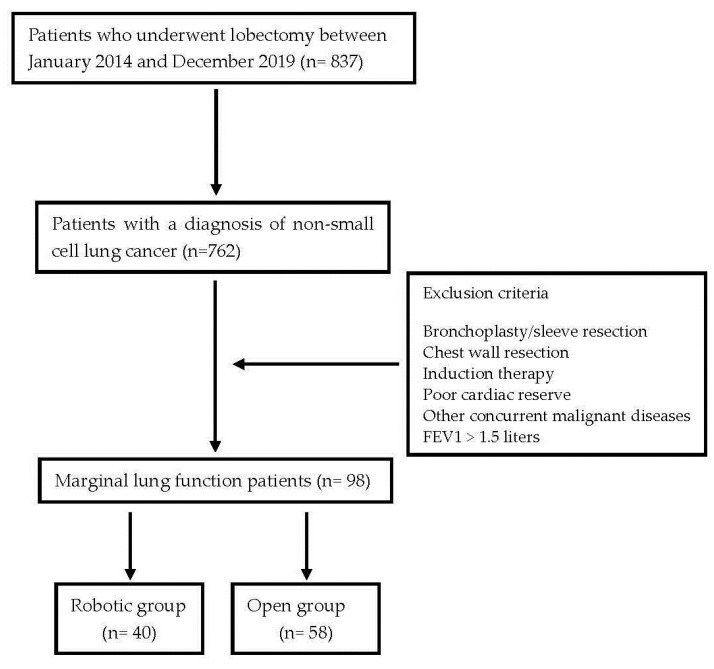
The study design.

Surgical procedures were performed using either the open technique via thoracotomy or a totally endoscopic 4-ports robotic approach using the da Vinci surgical system Si or Xi^®^ (Intuitive, Sunnyvale, CA, USA). Figure 1 summarizes the study design. 

Patient demographics, clinical characteristics and operative and postoperative results were collected and analyzed. Data from patients who underwent robotic lobectomy were collected and compared with data from patients treated with open lobectomy.

Comorbidities were assessed using the Charlson–Deyo comorbidity score [11]. 

The preoperative evaluation was based on a total body computed tomography (CT) scan with contrast enhancement and fluorine-18 fluorodeoxyglucose positron emission tomography-computed tomography (18F-FDG PET-CT). Invasive mediastinal lymph node evaluation, conducted via endobronchial ultrasound (EBUS) or surgical biopsy, was performed in instances of central tumors, enlarged lymph nodes on CT or nodes displaying positivity on 18F-FDG PET-CT.

Preoperatively, all patients underwent respiratory functional tests, cardiological evaluation, blood tests and anesthetic assessment to define the ASA (American Society of Anesthesiologists’ Classification of Physical Health) score. 

Postoperative complications were collected based on the Clavien–Dindo Complications Classification (CDCC) [12]. 

Postoperative management was similar in the two groups in terms of pain, chest drainage, urinary catheterization, mobilization, respiratory physiotherapy and diet.

Perioperative mortality was calculated as any death occurring within the first 30 days after surgery.

The calculation of 90-day mortality pertains to any death occurring within the first 90 days after surgery.

### Statistical Analysis

Categorical data were presented as absolute and relative (%) frequencies, and continuous data were summarized using the median and interquartile range (IQR).

To compare the “Surgical group” (open, robotic) with categorical and continuous factors, chi-square tests and Mann–Whitney tests were performed, respectively.

To compare the “Surgical group” (open, robotic 2014–2105, robotic 2016–2019) with categorical and continuous factors, chi-square tests and Kruskal Wallis tests, followed by multiple comparisons with the Bonferroni method, were performed, respectively. 

Furthermore, the “Surgical group” was compared with outcomes, such as air leak, complications, complication degree, length of stay and drainage duration, using multivariate logistic (using the step-wise method) and linear models, adjusting for confounding factors. Significance was fixed at 0.05 and all analyses were carried out using SPSS v.28 technology.

## 3. Results

### 3.1. General Aspects 

From January 2014 to December 2019, a total of 836 patients underwent lobectomy for lung cancer. Among them, 359 (43%) had the procedure performed using the robotic technique and 477 (57%) underwent the open approach. 

We analyzed the outcomes of the patients with marginal pulmonary function who underwent lobectomy, comparing the characteristics and results of the patients treated with robotic surgery to those treated with lateral or postero-lateral thoracotomy. 

The operations were performed by an expert team of surgeons and the robotic lobectomies were carried out by surgeons who also performed surgical procedures using the open approach.

In the robotic group, 40 (11%) patients with impaired lung function were included, whereas 58 (12%) patients were included in the open group. The demographics and clinical characteristics of the patients are summarized in Table 1. The patients in both groups were comparable in terms of their age, respiratory function, BMI (body mass index), ASA score and comorbidities. The robotic group included a higher number of adenocarcinomas and early-stage NSCLCs compared to the open group.

The operative and postoperative outcomes are summarized in Table 2. 

The robotic lobectomy method was characterized by a longer operative time than the open lobectomy method (mean 231 min vs. 128 min). During the surgical procedure, an intraoperative complication was observed in three cases: two (5%) cases experienced intraoperative bleeding in the robotic group and one (1.7%) case experienced intraoperative bleeding in the open group. Furthermore, the patients treated using the robotic approach had a shorter average postoperative stay (7.43 vs. 8.69 days), but no differences were found between the two groups in terms of the duration of drainage (6.55 vs. 6.43 days). 

A higher rate of postoperative complications was observed in the open group (44.8% vs. 37.5%). The open lobectomy group had a higher rate of anemia, which required a blood transfusion (15.5% vs. 5%), and atrial fibrillation (10% vs. 5%) and a lower rate of prolonged air leakage (12% vs. 25%).

No deaths occurred in either group within thirty days after the surgical procedure. Regarding the 90-day mortality, one (2.5%) patient died due to heart failure in the robotic group and two (3.4%) patients died in the open group, one for acute respiratory failure and the other for massive pulmonary embolism. 

### 3.2. Robotic vs. Open Surgery

The evaluation considered the influence of clinical, demographic and tumor features in comparing the two surgical groups (Table 3). To compare categorical and continuous factors between the robotic and open groups, chi-square tests and Mann–Whitney tests were performed, respectively. These analyses identified “gender” and “upper lobectomy” as confounding factors. 

In the multivariate analysis, patients with marginal pulmonary function who underwent robotic lobectomy had similar outcomes to those who were treated with open surgery (Table 4). 

### 3.3. Si da Vinci Surgical System vs. Xi da Vinci Surgical System

As previously described, the da Vinci Surgical System Si was used to perform the lobectomy surgeries from 2014 to 2015, whereas the da Vinci Surgical System Xi was used from 2016 onwards. We evaluated the possible impact of the different robotic surgical platforms on the outcomes, comparing them to the open technique (Table 5). 

To compare categorical and continuous factors between the open, Si da Vinci system and Xi da Vinci system groups, chi-square tests and Kruskal Wallis tests, followed by multiple comparisons using the Bonferroni method, were performed, respectively, and no confounding factors were found.

**Figure 2:**
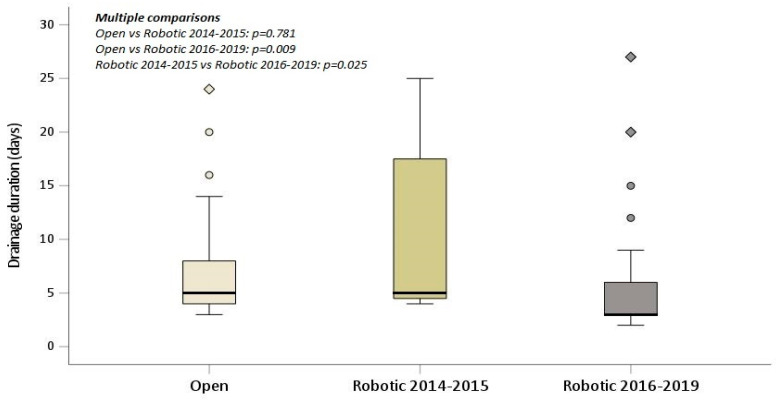
Drainage duration box plot displaying statistical significance for the three-group study.

In the multivariate analysis, robotic lobectomy performed using the Xi da Vinci Surgical System was independently associated with a lower postoperative complication rate and a statistically significant shorter duration of drainage (*p*-value 0.003) in the treatment of patients with marginal pulmonary function (Table 6). Figure 2 depicts a box plot illustrating the statistically significant difference in the duration of drainage for the three surgical groups.

## 4. Discussion

Although alternative strategies have been proposed over the years, lobectomy is still considered the gold standard treatment for early-stage NSCLC, offering a higher overall survival rate [13]. 

For NSCLC patients, the goal of surgical treatment is to successfully perform a radical oncological procedure involving pulmonary resection and hilar-mediastinal lymphadenectomy. This aims to ensure fast recovery and a satisfactory mental and physical postoperative quality of life for patients. Therefore, evaluating the preoperative clinical condition is fundamental for planning adequate operations and minimizing the risk of postoperative complications.

A series of diagnostic tests is recommended to assess the perioperative risk of patients eligible for lung resection. These tests typically encompass pulmonary function tests and cardiac examinations. Among these tests, spirometry assessing FEV1 and predicted postoperative (ppo) FEV1, along with evaluation of the lung’s diffusion capacity for carbon monoxide (DLCO) and ppoDLCO, play a globally recognized paramount role in evaluating lung function [4]. Over the years, studies have investigated the application of spirometry in the evaluation of patients after lung surgery. These studies have shown that the FEV1 value is strongly related to the development of postoperative complications and is considered the best single predictive factor of lung complications. However, the prognostic value of ppoFEV1 and ppoDLCO remains controversial [5]. Previous studies have described the association between DLCO < 60% and postoperative mortality, highlighting its role as a prognostic factor for overall survival after surgical resection. Furthermore, FEV1 is the parameter most commonly employed in clinical practice. Patients with an FEV1 > 1.5 liters are eligible for lobectomy with an average operative risk [14,15,16].

In patients with limited respiratory function, different local therapeutic options are available, including sublobar resection, stereotaxic radiotherapy, cryoablation and microwave or radiofrequency ablation. The above-mentioned options are suitable for preserving healthy lung tissue and preventing impairment to the patient’s physical state despite the lack of oncological radicality [17,18,19]. Sublobar resection is the alternative surgical choice to lobectomy for high-risk patients, characterized by marginal pulmonary function, advanced age or other severe comorbidities [20]. However, lobectomy represents the only applicable surgical option in some cases due to the characteristics of the neoplasm, for example, in large or central lesions. Furthermore, limited resection yields similar oncological outcomes compared to lobectomy only in selected early-stage NSCLC patients, representing a therapeutic compromise aimed at reducing the risk of perioperative complications in the majority of the high-risk patients [21,22].

The literature reports a clear and direct correlation between marginal pulmonary function and the incidence of complications in patients who have undergone lung resection with thoracotomy. These patients also required prolonged hospitalization and exhibited lower overall survival [23]. In addition, the development of postoperative complications has a substantial impact on the prognosis after pulmonary resection, representing a negative prognostic factor for survival among lung cancer patients [24,25]. Consequently, the treatment choice in patients with limited respiratory function is greatly dependent on the risk–benefit balance. The evaluation conducted by the multidisciplinary tumor board has become fundamental for determining the appropriate therapeutic approach.

Nevertheless, the introduction of minimally invasive surgery has modified the effect of surgery on patients with limited lung function. The reduction in trauma achieved through minimally invasive approaches preserves the chest wall respiratory mechanisms and decreases postoperative pain, facilitating a beneficial recovery [26]. Given this, guidelines recommend minimally invasive surgery for patients with marginal respiratory function in order to decrease the risk of mortality and morbidity [27]. 

In a comparison between open surgery and video-assisted thoracoscopic surgery (VATS) for anatomical lung resection in 70 patients with limited pulmonary reserve, thoracoscopic resection appeared to be associated with shorter hospital and intensive care stays and a lower incidence of pneumonia [28]. Similarly, Lau described a reduction in the length of hospital stay and the postoperative complication rate when evaluating patients with poor lung function who underwent thoracoscopic anatomical pulmonary resection [29]. Ceppa et al. also observed an increasing incidence of lung complications related to the reduction of predicted FEV1 in a large series of patients who underwent segmentectomy or lobectomy via thoracotomy, unlike patients treated with a thoracoscopic approach [30]. Similar results were reported by Yendamuri et al. in their evaluation of outcomes among patients with limited lung function. They observed a lower rate of morbidity and mortality after VATS lobectomy. The authors further suggest smoking cessation, effective pain control and pulmonary rehabilitation to optimize the postoperative outcomes [31].

After the surge in robotic surgery and its expanded use in the thoracic field, an extension of its surgical application to high-risk patients was observed. Due to its minimal invasiveness, robotic surgery minimizes trauma to the chest wall, reducing its impact on restricting mobility during breathing movements and postoperative pain, similar to what happens with VATS. Conversely, the insufflation of carbon dioxide that is usually required during robotic procedures to increase the intrathoracic space could impact the delicate balance of marginal pulmonary function patients, limiting function of the contralateral lung and leading to hypercapnia. Moreover, the longer operative time associated with docking and undocking the robotic system could cause respiratory complications, although this phenomenon has typically been described after open surgery and can be prevented using an anesthetic lung protective strategy [32].

Kneuertz observed considerable benefits in short-term postoperative outcomes were after robotic lobectomy. In this study, the postoperative outcomes of a subgroup of patients with a preoperative FEV1 or DLCO < 60% who underwent robotic and open lobectomy were compared. The results revealed a reduced length of hospital stay and a lower incidence of pulmonary complications were observed, particularly prolonged air leaks and pneumonia, after robotic lobectomy [33].

In our study, we evaluated the post-lobectomy outcomes of patients with a preoperative FEV1 < 1.5 liters who were treated using robotic or open surgery. This study focused on analyzing patients with a reduced FEV1, considering the role of FEV1 on postoperative complications according to the literature. This study assumed similar long-term results for the two different surgical approaches. The sample was characterized by significantly poor lung function. Specifically, the mean preoperative FEV1 was 1.25 liters in the robotic group and 1.26 liters in the open group. Additionally, the rate of current smoker patients was 43% in the robotic group and 38% in the open group. The patients in the two groups exhibited a similar duration of drainage and length of hospital stay, which was likely a consequence of the inherent frailty of high-risk patients. A lower incidence of grade II and III postoperative complications was observed in the robotic group (22.5% versus 32.8%). The patients who underwent robotic lobectomy exhibited a higher rate of prolonged air leaks and a lower incidence of other respiratory complications, anemia requiring blood transfusion and atrial fibrillation. Prolonged air leaks were observed in 25% of patients after robotic surgery and 12% of patients after open lobectomy. We observed a higher operative time and a lower postoperative complication rate in the robotic group, which was in line with other previous studies conducted on patients who were treated using robotic lobectomy [34,35]. In 2018, Kneuertz described an increased risk of prolonged air leaks due to the lack of tactile feedback during manipulation of the lung during robotic procedures, a risk which increases in cases involving emphysematous parenchyma in patients with a history of smoking and in those with marginal lung function [33]. In our previous study, we evaluated the application of robotic surgery for the treatment of NSCLC in patients affected by moderate or severe chronic obstructive pulmonary disease (COPD). A longer hospital stay and a higher rate of postoperative complications were observed in COPD patients when compared with other high-risk patients. In addition, prolonged air leaks were recorded in 54.5% of patients in the COPD group, in contrast to 24.1% in the other high-risk patients [36].

In the analysis of a sample of patients with a predicted preoperative FEV1 < 35% performed by Linden et al., prolonged air leak was the most common complication, recorded in 22% of patients [37]. This is in line with our results, in which prolonged air leaks complicated the postoperative hospital stay in 17.3% of the cases.

In our analysis, we evaluated the possible impact of different robotic platforms (da Vinci Si and Xi), characterized by different technologies, on postoperative outcomes. Robotic lobectomy performed with the Xi da Vinci Surgical System was independently associated with a statistically significant shorter duration of drainage and a lower postoperative complication rate. Moreover, the incidence of prolonged air leaks was remarkably reduced in robotic lobectomy performed with the latest da Vinci platform, suggesting the role of increased surgical expertise and advancements in the technological features in enhancing surgical outcomes. 

The monocentric and retrospective nature of this study is a limitation. A comparison with the outcomes of VATS lobectomy was not possible due to the early introduction of robotic surgery in our hospital in 2001, leading to restrictions in the utilization of thoracoscopic surgery. Further prospective randomized studies are necessary to confirm these results.

## 5. Conclusions

Robotic lobectomy is a safe and feasible procedure for patients with poor pulmonary function. Moreover, the evolution of technology in the robotic system associated with high-volume surgical activity can improve the postoperative outcomes of high-risk patients, including those with marginal pulmonary function.

## Figures and Tables

**Table 1:** Demographics and clinical characteristics of marginal pulmonary function patients undergoing robotic and open lobectomy. Statistics: absolute frequency (%) or mean (range).

	Robotic Lobectomy (n = 40)	Open Lobectomy (n = 58)
Age (years)	73 (60–83)	71 (51–85)
Male	10 (25)	26 (45)
History of smoking	32 (80)	50 (86)
Current smoker	17 (43)	22 (38)
Former smoker	15 (38)	28 (48)
ASA score		
I	0 (0)	0 (0)
II	16 (40)	16 (28)
III	24 (60)	41 (72)
BMI (kg/m)	25 (17–39)	27 (17–43)
FEV1 value (L)	1.25 (0.80–1.50)	1.26 (0.76–1.50)
Predicted FEV1 (%)	69 (42–123)	62 (31–115)
FEV1/FVC value (%)	60 (34–91)	60 (34–89)
Ppo FEV1 (%)	53 (32–107)	49 (25–89)
Comorbidities		
Presence	39 (98)	53 (91)
COPD	14 (35)	23 (40)
Cardiovascular disease	6 (15)	11 (20)
Hypertension	6 (15)	7 (12)
Diabetes	2 (5)	5 (9)
Others	11 (28)	7 (12)
Pathological stage		
I	26 (65)	23 (40)
II	9 (23)	22 (38)
III	3 (8)	10 (17)
IV	0 (0)	2 (3)
Histopathology		
Adenocarcinoma	21 (53)	24 (41)
Squamous carcinoma	11 (28)	22 (38)
Others	8 (20)	12 (21)

Abbreviations: ASA (American Society of Anesthesiologists’ Classification of Physical Health); BMI (body mass index); COPD (chronic obstructive pulmonary disease); FEV1 (forced expiratory volume in the first second); FVC (forced vital capacity); Ppo FEV1 (predicted postoperative forced expiratory volume at one second).

**Table 2:** General surgical and postoperative outcomes. Statistics: absolute frequency (%) or mean (range).

	Robotic Lobectomy (n = 40)	Open Lobectomy (n = 58)
Surgical time (min)	231 (130–415)	128 (50–235)
Laterality		
Right	28 (70)	34 (59)
Left	12 (30)	24 (41)
Type of lobectomy		
Upper right lobectomy	9 (22.5)	23 (39.7)
Upper left lobectomy	6 (15)	13 (22.4)
Middle lobectomy	8 (20)	5 (8.6)
Lower right lobectomy	11 (27.5)	6 (10.3)
Lower left lobectomy	6 (15)	11 (19)
Intraoperative complications	2 (5)	1 (2)
Hospital stay (days)	7.43 (3–23)	8.69 (4–24)
Drainage duration (days)	6.55 (2–27)	6.43 (3–24)
Postoperative complications	15 (37.5)	26 (44.8)
Complication grade according to CDCC		
I	6 (15)	7 (12)
II	4 (10)	15 (25.9)
III	5 (12.5)	4 (6.9)
Type of complication	10 (25)	7 (12)
Prolonged air leakAtrial Fibrillation	2 (5)	6 (10.3)
Anemization with blood transfusion	2 (5)	9 (15.5)
Atelectasis		1 (1.7)
Respiratory failure		1 (1.7)
Other pulmonary event	1 (2.5)	2 (3.4)
30-day Mortality	0 (0)	0 (0)
90-day Mortality	1 (2.5)	2 (3.4)

Abbreviations: CDCC (Clavien–Dindo Complications Classification).

**Table 3:** Influence of clinical, demographic and tumor factors on the “Surgical group” (open, robotic). Statistics: absolute frequency (%) or median (IQR).

Factor	Open	Robotic	*p*-Value
Age	73 (67–76)	72.5 (69–76)	0.690
Gender			0.045
M	26	10	
F	32	30	
BMI	26.4 (22–30.9)	24.8 (20.1–27.8)	0.089
ASA	3 (2–3)	3 (2–3)	0.169
FEV1	1.3 (1.1–1.4)	1.3 (1.2–1.4)	0.553
FEV1/FVC	0.58 (0.49–0.73)	0.62 (0.48–0.72)	0.980
History of smoking			0.520
No	8	8	
Former smoker	28	15	
Current smoker	22	17	
Histology			0.495
Other	18	8	
Squamous	22	11	
Adenocarcinoma	24	21	
Lower lobectomy			0.178
No	41	23	
Yes	17	17	
Middle lobectomy			0.103
No	53	32	
Yes	5	8	
Upper lobectomy			0.017
No	22	25	
Yes	36	15	
Laterality			0.251
Right	34	28	
Left	24	12	

Abbreviations: ASA (American Society of Anesthesiologists’ Classification of Physical Health); BMI (body mass index); FEV1 (forced expiratory volume in the first second); FVC (forced vital capacity).

**Table 4:** Comparison between “Surgical group” and outcomes using multivariate models adjusted for “robotic surgery 2014–2015”, “gender” and “upper lobe”. Statistics: *p*-value.

Outcome	Multivariate MODEL	*p*-Value Related to “Surgical group”
Prolonged air leak (0) no (1) yes	Logistic	0.097
Complication (0) no (1) yes	Logistic	0.470
Complication degree (range: 0–3)	Linear	0.397
Hospital stay (days)	Linear	0.398
Drainage duration (days)	Linear	0.780

**Table 5:** Influence of clinical, demographic and tumor factors on the “Surgical group” (open, robotic 2014–2015, robotic 2016–2019). Statistics: absolute frequency (%) or median (IQR).

Factor	Open	Si System (2014–2015)	Xi System (2016–2019)	*p*-Value
Age	73 (67–76)	73 (73–76)	72 (69–75)	0.636
Gender				0.109
M	26 (44.8)	1 (14.3)	9 (27.3)	
F	32 (55.2)	6 (85.7)	24 (72.7)	
BMI	26.5 (22–30.9)	23.4 (22.1–24.8)	25 (20.1–28.9)	0.163
ASA	3 (2–3)	2 (2–2.5)	3 (2–3)	0.064
FEV1	1.31 (1.14–1.42)	1.26 (1.09–1.37)	1.28 (1.18–1.37)	0.758
FEV1/FVC	0.59 (0.49–0.73)	0.67 (0.62–0.73)	0.60 (0.48–0.69)	0.670
History of smoking				0.764
No	8 (13.8)	2 (28.6)	6 (18.2)	
Former smoker	28 (48.3)	2 (28.6)	13 (39.4)	
Current smoker	22 (37.9)	3 (42.9)	14 (42.4)	
Histology				0.499
Other	12 (20.7)	2 (28.6)	6 (18.2)	
Squamous	22 (37.9)	3 (42.9)	8 (24.2)	
Adenocarcinoma	24 (41.4)	2 28.6)	19 (57.6)	
Type of lobectomy				0.144
Lower lobectomy	17 (29.3)	3 (42.9)	14 (42.4)	
Upper lobectomy	36 (62.1)	2 (28.6)	13 (39.4)	
Middle lobectomy	5 (8.6)	2 (28.6)	6 (18.2)	
Laterality				0.515
Right	34 (58.6)	5 (71.4)	23 (69.7)	
Left	24 (41.4)	2 (28.6)	10 (30.3)	

Abbreviations: ASA (American Society of Anesthesiologists’ Classification of Physical Health); BMI (body mass index); FEV1 (forced expiratory volume in the first second); FVC (forced vital capacity).

**Table 6:** Comparison between “Surgical group” and outcomes. Statistics: absolute frequency (%) or median (IQR).

Outcome	Open	Si System (2014–2015)	Xi System (2016–2019)	*p*-Value
Prolonged air leak				0.098
No	51 (87.9)	4 (57.1)	26 (78.8)	
Yes	7 (12.1)	3 (42.9)	7 (21.2)	
Complication				0.393
No	32 (55.2)	3 (42.9)	22 (66.7)	
Yes	26 (44.8)	4 (57.1)	11 (33.3)	
Complication degree	0 (0–2)	1 (0–2.5)	0 (0–1)	0.292
Length of stay	7 (5–9)	6 (5–9)	6 (4–8)	0.074
Drainage duration	5 (4–8)	5 (4.5–17.5)	3 (3-6)	0.003

## Data Availability

The data presented in this study are available on request from the corresponding author. The data are not publicly available due to privacy reasons.
